# Strategic pathways to International Classification of Diseases, 11th Revision, adoption in France and the United States

**DOI:** 10.1093/haschl/qxaf099

**Published:** 2025-07-01

**Authors:** Bastien Boussat, Robert Jakob, Laurent Boyer, Patrick S Romano

**Affiliations:** Department of Clinical Epidemiology, Grenoble Alps University, Grenoble 38000, France; O’Brien Institute for Public Health, University of Calgary, Calgary, AB, Canada T2N 1N4; Classifications and Terminologies Unit, World Health Organization (WHO), Geneva CH-1211, Switzerland; CEReSS-Health Service Research and Quality of Life Center, Assistance Publique-Hôpitaux de Marseille, Aix-Marseille University, Marseille 13005, France; Division of General Medicine and Center for Healthcare Policy and Research, University of California, Davis, Sacramento, CA 95817, United States

**Keywords:** ICD-11, health classification, France, United States, ATIH, NCVHS, healthcare innovation, WHO-FIC, health interoperability, public health, coding tools, digital health, healthcare research, pilot testing, system transition

## Abstract

The International Classification of Diseases, 11th Revision (ICD-11), developed by the World Health Organization, represents a transformative update to global health data classification systems. Building on the foundation of ICD-10, it introduces innovative features such as multilingual coding, advanced interoperability, postcoordination, and improved specificity, enabling better alignment with modern healthcare and digital information systems. This commentary explores the adoption pathways for ICD-11 in France and the United States, 2 countries with complex healthcare infrastructures and distinct implementation strategies. France's phased roadmap, led by the National Health Information Agency, prioritizes system readiness, workforce training, and pilot testing to ensure smooth integration with hospital information systems. In contrast, the United States, guided by the National Committee on Vital and Health Statistics, focuses on regulatory alignment, funding models, and system modifications to support a seamless transition. The manuscript underscores the critical role of academic research in refining ICD-11's applications, assessing its impact on healthcare quality, and optimizing tools for implementation. Drawing lessons from early adopters globally, we advocate for a coordinated, resource-driven approach to achieve full ICD-11 adoption within 5 years. This transition is poised to enhance health data accuracy, support clinical research, and improve population health outcomes worldwide.

## Introduction

The International Classification of Diseases, 11th Revision (ICD-11), developed by the World Health Organization (WHO), represents the most recent global standard for classifying health conditions.^1^ Building upon over a century of previous classifications, ICD-11, released in 2018, reflects extensive innovations that support the needs of modern healthcare and digital information systems.^2^ Specifically, it introduces multilingual coding, advanced interoperability and tooling, postcoordination of clinical concepts, and enhanced specificity compared with ICD-10 and its clinical modifications, such as International Classification of Diseases, 10th Revision, Clinical Modification (ICD-10-CM) in the United States^3^ (Table 1).

**Table 1.** New features and advantages of ICD-11.

Aspect	Description
ICD-11: more than a new code	ICD-11 offers a substantial upgrade over ICD-10 and its clinical modifications, such as ICD-10-CM in the United States, by incorporating multiple new features and capabilities designed to meet modern healthcare needs.
Expanded coding specificity and postcoordination	One of the most transformative aspects of ICD-11 is its postcoordination system, which allows users to combine codes to describe clinical details with high precision. This flexibility enables the capture of complex diagnoses and comorbidities by adding qualifiers (eg, severity, laterality, anatomic location, or clinical manifestations), thereby conveying a complete clinical picture and avoiding the proliferation of precoordinated codes seen in ICD-10-CM.
Digital compatibility	Unlike ICD-10, ICD-11 is designed for seamless integration with EHRs and other digital health platforms. It provides an API that enables healthcare systems to directly incorporate its codes and structure, supporting interoperability, real-time data sharing, analysis, and integration into decision support tools.
Global standardization and adaptability	While ICD-11 establishes a standardized framework for international health data, it also offers the flexibility for adaptation to various healthcare settings. Its design allows countries to develop linearizations that meet their national needs without losing international comparability, which is critical for coordinated public health responses, global health research, and health informatics.
Comprehensive data granularity	The depth of ICD-11's semantic architecture, built on a carefully curated Foundation, enables an unprecedented level of detail in capturing health data. This granularity is key for precision medicine, clinical and population health research, and policy planning.

Abbreviations: API, Application Programming Interface; EHRs, electronic health records; EHRICD-11, International Classification of Diseases, 11th Revision.

Recent WHO reports indicate that a total of 132 Member States and areas are at various phases of implementing the new classification system. Specifically, 72 countries have commenced the implementation process (including translation efforts), 50 countries are conducting or expanding implementation pilots, and 14 countries and areas have already begun collecting or reporting data using ICD-11 coding.^4^

The transition from ICD-10 to ICD-11 will streamline health reporting, foster uniformity across countries, and integrate modern health challenges such as antimicrobial resistance, patient safety, and rare diseases. Accordingly, the adoption of ICD-11 across healthcare systems worldwide promises to improve disease tracking, enhance healthcare interoperability, and support population health research. However, the full transition to ICD-11 requires coordinated efforts, especially in countries with complex healthcare infrastructure like France and the United States.^5^ In both France and the United States, initiatives are underway to adopt ICD-11, and these efforts need strong support and clear strategic goals to achieve full success within 5 years.^6^

## Progress in France and the United States: where are we?

### France: a strategic approach

In France, the National Health Information Agency (ATIH) has assumed a leading role in planning and coordinating ICD-11 implementation efforts. Through ATIH's leadership, France has laid out a structured, multi-phase roadmap for ICD-11 adoption that includes system readiness, workforce training, and ongoing evaluation to ensure interoperability within the French health information infrastructure. Unlike in the United States, one of the initial challenges in France was the complete translation of the ICD-11 classification and its methodological guides into French—a process now fully completed. An impact study conducted in 2023 concluded that the implementation project should be managed in close collaboration with all key stakeholders (including national agencies and the Ministry of Health).^7^

Key aspects of ATIH's approach include partnerships with hospital information system providers to integrate ICD-11 coding tools and compatibility updates. Early stages of the roadmap focus on creating robust guidance for ICD-11 use and preparing dual-coding tools to allow health professionals to transition smoothly and to minimize disruption to routine coding practices.^5^ In 2025, a competitive call for pilot projects has been launched targeting pilot double coding in 10 major university hospitals, aiming to code ∼10 000 hospital stays across medicine, surgery, and obstetrics, with additional pilot projects planned for 2026 and 2027.^8^ These initiatives are designed not only to prepare clinical coders but also to provide precise estimates of the financial investment required (eg, for professional training and for integrating the WHO Application Programming Interface [API] coding tool into hospital information systems). France's experience illustrates a proactive approach, although sustained investment and policy commitment are essential to meet the full implementation target.

### United States: cross-sector engagement

In the United States, the transition to ICD-11 is also gathering momentum, though the structure and strategy differ from those in France. Under the National Committee on Vital and Health Statistics (NCVHS), the United States established a dedicated ICD-11 Workgroup tasked with assessing the feasibility, regulatory considerations, and resource needs for adoption.^9^ As a multi-stakeholder body, the NCVHS facilitated partnerships across federal agencies, healthcare providers, and industry representatives to tackle the specific challenges that ICD-11 presents for the US health system, especially with regard to existing statutory and regulatory frameworks. The NCVHS prioritized timely and well-informed action so that the United States can avoid repeating the costs and burdens associated with its delayed implementation of ICD-10 for morbidity reporting, including the development of a full US-specific clinical modification as occurred for ICD-10.

The ICD-11 Workgroup process focused on information gathering by reviewing research studies and findings, soliciting input from subject matter experts, and engaging all key stakeholders.^10^ One focus of the US initiative was to explore how ICD-11 could impact payment models, particularly diagnosis-related groups and other billing and authorization processes, which directly influence payment under Medicare and other payers. Transitioning to ICD-11 is expected to enable more precise categorization of patient conditions, thereby leading to more equitable reimbursement that aligns closely with actual patient complexity and resource utilization. By carefully assessing how ICD-10-CM is being used, and clarifying its strengths and limitations, the Workgroup sought to identify areas where ICD-11 might address these limitations, such as improving coding specificity, abandoning underused precoordinated codes, and enhancing compatibility with emerging health informatics platforms.^11^ However, the US effort also highlights the challenges of aligning the transition with existing policies and payment models, as the shift to ICD-11 will require extensive updates to electronic health record (EHR) systems, data reporting structures, and policies that govern authorization and payment for healthcare services.^2^ A dedicated stream of funding, extensive field testing, policy adjustments, and collaboration among all stakeholders will be essential for ensuring ICD-11's smooth adoption in the complex US healthcare landscape.

## The role of research

Academic research is indispensable for assessing the real-world impact of ICD-11 on healthcare quality and safety, refining its practical applications, and evaluating and enhancing associated tooling, including automated or semi-automated coding from free text. Research institutions can provide invaluable insights on interoperability, health informatics integration, and the validity of ICD-11's enhanced coding system. These studies are particularly valuable for evaluating how well ICD-11 supports clinical, epidemiological, and population health research, especially when compared with ICD-10 and ICD-10-CM. For example, the improved granularity of ICD-11 enables earlier outbreak detection by allowing public health authorities to monitor subtle shifts in disease patterns, while more accurate tracking of chronic conditions supports targeted interventions that reduce hospital readmissions and improve long-term disease management. Moreover, such research projects are critical in providing the evidence base needed to guide policy decisions and optimize resource allocation at both national and international levels. Enhanced data quality also supports more effective treatment protocols and resource allocation, thereby improving overall patient care and public health outcomes.

To support this activity, dedicated grants for ICD-11 research should be established. These grants would empower researchers to explore the intricacies of ICD-11, from data integration methods to optimizing coding accuracy, and to develop strategies that address gaps and streamline the transition. Collaborative research projects between academic and clinical settings can act as a bridge, allowing feedback from real-world applications to influence future ICD-11 enhancements. Independent research teams can play a pivotal role in advancing our understanding of ICD-11's impact, thereby guiding policy decisions. International funding mechanisms (such as Horizon Europe) could also support collaborative projects focused on aspects like international comparability, development of performance indicators, and the application of artificial intelligence in health data.

## Global collaboration: learning from international experience

While France and the United States are making strides, other countries, such as China, Iran, Kuwait, Malaysia, and the United Nations Relief and Works Agency for Palestine Refugees in the Near East, are further along in their ICD-11 adoption efforts.^12-16^ Early-stage implementation projects in diverse regions, unburdened by complex legacy systems, provide valuable insights into how ICD-11 can be implemented effectively to support universal health coverage and public health goals. Observations from these early adopters reinforce the importance of flexibility, adaptation to local contexts, and stakeholder engagement in navigating ICD-11 integration challenges.

The WHO Family of International Classifications (WHO-FIC) network, which connects national WHO collaborating centers, offers a crucial platform for sharing these insights.^7^ Through the WHO-FIC, member countries can access practical knowledge from their peers, adopt best practices, and collaborate on solutions to shared challenges, such as data mapping, workforce training, and enhancing coding accuracy.

In addition to knowledge exchange, WHO-FIC's collaborative model could be instrumental in establishing standardized guidelines and training resources. These tools support countries at all stages of ICD-11 implementation, ensuring that even smaller or less-resourced healthcare systems can benefit from ICD-11's potential.^5^ Leveraging the WHO-FIC network through regular international exchanges, collaborative research projects, and direct dialog among national agencies, technology vendors, and coding experts is essential to harmonize coding practices and overcome implementation challenges.

## Conclusion

The transition to ICD-11 is not without challenges, but the potential benefits are clear. With improved data precision and enhanced interoperability, ICD-11 is positioned to shape the future of healthcare on a global scale. To realize this vision, health authorities worldwide, along with collaborative networks such as WHO-FIC, must continue to support these initiatives and provide targeted resources to foster adoption. A realistic yet ambitious goal of full ICD-11 implementation within 5 years, with full deployment expected by ∼2030, is achievable through coordinated efforts at the national, international, and technical levels. In doing so, ICD-11 will strengthen public health, clinical research, and international health data standards in profound ways.

## Supplementary Material

qxaf099_Supplementary_Data
